# A DNA Vaccine in Which the RSV-F Ectodomain Is Covalently Linked to the *Burkholderia pseudomallei* Antigens TssM and Hcp1 Augments the Humoral and Cytotoxic Response in Mice

**DOI:** 10.3389/fimmu.2019.02411

**Published:** 2019-10-11

**Authors:** Victor Solodushko, Vira Bitko, Robert Barrington, Brian Fouty

**Affiliations:** ^1^Department of Pharmacology, University of South Alabama School of Medicine, Mobile, AL, United States; ^2^Center for Lung Biology, University of South Alabama School of Medicine, Mobile, AL, United States; ^3^Emergent BioSolutions, Gaithersburg, MD, United States; ^4^Department of Microbiology and Immunology, University of South Alabama School of Medicine, Mobile, AL, United States; ^5^Department of Internal Medicine, University of South Alabama School of Medicine, Mobile, AL, United States

**Keywords:** DNA vaccine, RSV-F, melioidosis, antibody response, cytotoxicity

## Abstract

DNA vaccines have great potential to control infectious disease, particularly those caused by intracellular organisms. They are inexpensive to produce and can be quickly modified to combat emerging infectious threats, but often fail to generate a strong immunologic response limiting enthusiasm for their use in humans and animals. To improve the immunogenic response, we developed a DNA vaccine in which the F protein ectodomain of Respiratory Syncytial Virus (RSV-F) was covalently linked to specific antigens of interest. The presence of the RSV-F ectodomain allowed secretion of the translated fusion product out of the originally transfected cells followed by its active binding to adjacent cells. This allowed the targeting of a greater number of cells than those originally transfected, enhancing both humoral and cytotoxic immune responses against the expressed antigen(s). We developed an engrafted mouse model that used antigen-expressing tumor cells to assess the *in vivo* cytotoxic immune response to specific antigens. We then used this model to demonstrate that a DNA vaccine in which the RSV-F ectodomain is fused to two antigens expressed by *Burkholderia pseudomallei*, the intracellular gram-negative organism that causes melioidosis, generated a stronger cytotoxic response than a DNA vaccine that lacked the RSV-F sequence while still generating a robust humoral response.

## Introduction

DNA vaccines have great potential for treating infectious and non-infectious diseases throughout the world (1). They are inexpensive to produce and can be easily modified to accommodate a variety of targets. In addition, DNA vaccines offer the greatest likelihood of protecting against intracellular infectious organisms (2–6). This is because they not only provide intracellular and membrane antigen delivery, but they also maintain a prolonged duration of antigen presentation to the immune system, thus directing T lymphocytes toward proliferation rather than toward an early conversion into memory cells (7, 8).

One of the major limitations of current DNA vaccines has been their frequent failure to generate a strong immune response because they do not target (transfect) enough cells during the initial vaccination. This leads to antigen expression on only a few cells (those that were initially transfected) which provides an insufficient signal to stimulate a strong humoral and cytotoxic immune response (9).

Human respiratory syncytial virus (RSV), a member of the *Paramyxoviridae* family, is a major cause of severe acute lower respiratory tract infection in infants, the elderly, and immunocompromised adults (10). The F protein of RSV (RSV-F) is a glycosylated surface protein that is critical in facilitating fusion of RSV to the host cell membrane (11). If folded correctly, RSV-F forms trimers and is considered metastable (12–15). Shortly after viral secretion, RSV-F undergoes triggering (the translocation of the hydrophobic tail from within the hydrophilic region of RSV-F to outside the region) followed by a series of dramatic structural rearrangements that result in the insertion of the fusion peptide into the target cell membrane (13). In live cells, this anchoring activates clathrin-mediated endocytosis (16). A soluble form of RSV-F, in which the transmembrane and cytoplasmic domains were replaced with molecular tags retained the ability to form trimers in its pre-fusion (pre-triggered) state after secretion and fuse to target membranes after triggering (12).

In an attempt to overcome the limited antigen presentation of current DNA vaccines, we proposed to link a coding region of the RSV-F ectodomain to the coding region of immunogenic antigens of interest. The rationale was that in initially transfected cells, translation of the DNA vaccine would produce a protein (polypeptide) in which the antigen(s) of interest were covalently linked to an extracellular domain of RSV-F. The presence of this RSV-F sequence would allow secretion of the entire fusion protein from the cell followed by its attachment to the membranes of other neighboring non-immune and, more importantly, immune cells that were not initially transfected; once internalized and processed, the fusion protein would then be presented by these cells. This should induce a stronger humoral and cytotoxic immune response to the encoded antigens.

We tested the efficacy of this fusion DNA vaccine using antigens expressed by *Burkholderia pseudomallei*, the intracellular gram-negative bacteria responsible for the disease melioidosis. Melioidosis is a world-wide problem of increasing importance (17). Endemic disease is more widespread than previously thought, with an epicenter in Southeast Asia, India, and northern Australia, but also with a higher than previously realized incidence in South and Central America (18, 19). Due to its ability to cause disease when inhaled, *B. pseudomallei* has been designated by the Center for Disease Control (CDC) as a Tier I potential select agent (19). At present, there is no effective vaccine against this organism.

The two selected *B. pseudomallei* antigens, Hcp1 (required for the assembly of the type 6 secretion system and the export of its effectors) and a modified immunogenic region of TssM (a deubiquitinase that inhibits the NF-κB and interferon-β pathways) (20–22), are conserved components of the virulence-associated *B. pseudomallei* type VI and type II secretion systems, respectively (23). Both antigens are expressed at relatively high levels in humans and animals during bacterial infection and were shown to induce a cytotoxic immune response in animal models of experimental melioidosis (21–26). We demonstrate that animals vaccinated with an experimental DNA vaccine in which RSV-F was linked to TssM and Hcp1 was able to generate a specific antibody response and more quickly clear cells expressing these antigens than animals vaccinated with a DNA vaccine encoding TssM and Hcp1 without RSV-F.

## Materials and Methods

### Materials

Dulbecco's Modified Eagle Medium (DMEM) with and without phenol red, 0.05% trypsin/0.53 mM EDTA and L-glutamine were all purchased from Gibco (Grand Island, NY). Fetal bovine serum (FBS) was purchased from Atlanta Biologicals (Lawrenceville, GA). Gentamicin Sulfate was from Corning (Corning, NY). TransIT-X2TM transfection reagent was purchased from Mirus (Madison, WI). FuGENE 6 and FuGENE HD Transfection Reagents were purchased from Roche Diagnostics (Indianapolis, IN). All restriction enzymes, DNA polymerase I (Klenow) and High Efficiency Competent *E. Coli* Cells [NEB 10-beta; Cat. No: C3019H] were from New England BioLabs (Ipswich, MA). Hi-Lo DNA Markers were from Minnesota Molecular, Inc. (Cat. No: 1010, Minneapolis, MN). Dimethyl Sulphoxide (DMSO) and tetramethylbenzidine were purchased from Sigma-Aldrich (St. Louis, MO). DNeasy Blood and Tissue and RNeasy Mini kits were from Qiagen (Germantown, MD). USB VeriQuest Fast SYBR Green qPCR Master Mix with Fluorescein Kit was from Afflymetrix (Santa Clara, CA). SuperSignal West Dura was purchased from Pierce (Rockford, IL). Enzyme-free Cell Dissociation Buffer, was from ThermoFisher Scientific (Waltham, Massachusetts). NucBlue Live Cell Stain (Cat. No: R37605) was from Invitrogen (Carlsbad, CA).

### Antibodies

Goat Anti-Mouse IgG_1_, Human ads-HRP (Cat. No: 1070-05) and Goat Anti-Mouse IgG_2a_, Human ads-HRP (Cat. No: 1080-05) were from SouthernBiotech (Birmingham, AL). Mouse monoclonal anti-RSV antibody (2F7) (Cat. No: sc-101362), mouse anti-rabbit IgG conjugated to HRP (Cat. No: sc-2357), mouse IgG kappa binding protein (m-IgGκ BP) conjugated to HRP (Cat. No: sc-516102) were from Santa Cruz (Dallas, TX). Sheep anti-mouse IgG conjugated to HRP (Cat. No: NA931-1ML) were from Sigma-Aldrich (St. Louis, MO). Anti-mCherry monoclonal antibody (16D7) conjugated to Alexa Fluor 488 (Cat. No: M11239) were from Invitrogen (Carlsbad, CA). Rabbit anti-mCherry polyclonal antibody (Cat. No: PA5-34974) were from ThermoFisher Scientific (Waltham, Massachusetts).

### Animals and Cells

Specific pathogen-free BALB/c(H-2^d^) mice were purchased from Charles River Laboratories (Kingston, NY) and housed at the University of South Alabama animal care unit at the appropriate biosafety level. The University of South Alabama Animal Care and Use Committee has approved all mouse studies under PHS assurance. For *in vivo* studies, six animals were included in each immunization group. CT26.WT cells, an undifferentiated colon carcinoma cell line that forms tumors when injected into immunocompetent BALB/c mice, were purchased from ATCC (ATCC CRL-2638) and used for *in vitro* and *in vivo* experiments in these studies. The cells were grown in humidified incubators in DMEM/10%FBS supplemented with Gentamicin Sulfate and L-glutamine at 37°C in 5% CO_2_ and routinely passaged after reaching 80% confluency. Cells were harvested by 0.05% trypsin/0.53 mM EDTA digestion and counted with Coulter Z1 (Coulter Electronics). Counts were made in triplicate.

### Flow Cytometry Analysis

Cells were transfected with plasmids using TransIT-X2TM (Mirus), FuGENE 6, or FuGENE HD transfection (Roche) reagent following the manufacturer's instructions (1 μg plasmid DNA per 1 × 10^6^ cells for the smallest vector in any experimental set and adjusted for other plasmids to keep the same molar concentration). Cells were harvested by 0.05% trypsin/0.53 mM EDTA digestion, washed, and analyzed or sorted using positive or negative selections for mCherry (ex./em. of 587/610 nm) and/or eGFP (ex./em. of 484/507 nm) fluorescence by BD Biosciences FACSAria cell sorter/analyzer in the University of South Alabama Flow Cytometry Core. For some experiments cells were re-seeded after sorting for the following experiments and the fluorescent marker(s)-positive/negative cells was monitored at later time points. For some experiments cells were immune-stained with the anti-mCherry monoclonal antibody (16D7) conjugated to Alexa Fluor 488 (Cat. No: M11239) prior to analysis.

### Immunofluorescent Microscopy

For live cell imaging, CT26.WT cells were seeded into 35 mm MatTek glass bottom dishes (Part No: P35G-1.5-10-C) in DMEM supplemented with 10% heat-inactivated fetal bovine serum and 2 mM L-Glutamine. After 24 h of culturing, cells were transfected with the control construct (mCherry only) or the test construct (RSVF-mCherry fusion). Cells were grown at 37C° and 5% CO_2_ for an additional 48 h before analysis. Live cells were stained with anti-mCherry monoclonal antibody conjugated to Alexa Fluor 488 (16D7) for 20 min and washed three times with the cultured medium. Two drops of NucBlue (Invitrogen) were applied to each well with the final wash and placed in a humidity-and temperature-controlled environment for 10 min. The medium was then replaced with PBS and samples analyzed with a Nikon Eclipse Ti microscope (Nikon). For NucBlue, a 375–390-nm excitation and a 420–490-nm emission filter were used. For mCherry, a 540–580-nm excitation and a 600–680-nm emission filter were used. For Alexa Fluor 488, a 450–490-nm excitation and a 500–540-nm emission filter were used.

### Cytokine Measurements in Serum

Cytokines in the serum of vaccinated and control mice were analyzed by a bead-based multiplex assay LEGENDPlex (BioLegend, San Diego, CA) for Th1/Th2 cytokine panel, according to manufacturer instructions using a FACSAria cell Flow Cytometer (BD Biosciences, San Jose, CA). Samples were analyzed using FlowJo (Ashland, Oregon) data analysis software.

### Quantitative PCR

Total DNA was isolated from cells using the Blood and Tissue Kit (Qiagen, Cat. No: 69504) according to the manufacturer's protocols using RNAse A for removal of contaminating RNA. Fast purification of high-quality RNA from cells was performed with RNeasy Mini kit (Qiagen, Cat No: 74104). Quantitative Real Time PCR was then performed using the USB VeriQuest Fast SYBR Green qPCR Master Mix with Fluorescein Kit (Afflymetrix) for DNA or RNA samples according to the protocol. All primers were designed by the Beacon program to amplify 100–250 bp sequences within the specific fragments of the experimental genes. Sequences for the 28S (housekeeping control) primers were TCGGCTCTTCCTATCATT (forward) and GCAACAACACATCATCAG (reverse). Sequences for the mCherry primers were GTCAAGACCACCTACAAG (forward) and ATGGTGTAGTCCTCGTTG (reverse). Sequences for the eGFP primers were GAACCGCATCGAGCTGAAGG (forward) and CGTTGTGGCTGTTGTAGTTGTAC (reverse). Sequences for the Hcp1 primers were AAAGTCCTCATAACACACA (forward) and CTATGTAGCCGAGAAGAC (reverse). Sequences for the TssM primers were CTTTGCGGATGCCACTTTAC (forward) and GGGAAGGTCTGCGTAGGA (reverse).

### Engineering CT26.WT Cells to Stably Express Antigen and/or Fluorescent Markers to Study Antibody Response and Cell Mediated Cytotoxicity in Mice

CT26.WT cells were stably modified to express Hcp1 and/or TssM antigens and one of two fluorescent protein reporter—mCherry or eGFP using the minimal piggyBac delivery system (27) based on pVAX1 plasmid backbone (Thermo Fisher Scientific). All vectors delivered transgene(s) under the control of a CMV promoter and their expression was terminated by the bovine growth hormone polyadenylation [bGH-Poly(A)] signal. One control vector delivered mCherry only. The next three bicistronic vectors expressed mCherry as the first gene and the *B. pseudomollei* antigen genes downstream of IRES. In one bicistronic vector we delivered the full-length protein Hcp1 (BPSS1498; *B. pseudomallei* K96243). The second bicistronic vector had an immunogenic region of TssM (BPSS1512; *B. pseudomallei* K96243; amino acids 191–474). The third bicistronic vector harbored both sequences separated by a flexible linker (Hcp1 and TssM fused together). Because the cysteine at position 102 in TssM is critical for its ability to evade the host immune system (26), we introduced a Cys to Gly mutation (i.e., Cys^102^ was replaced by Gly^102^) to block this immunosuppressive activity in all constructs harboring this truncated TssM gene. The second control vector delivered eGFP instead of mCherry. A total 5 × 10^5^ modified CT26.WT cells were injected subcutaneously into mouse-tails in combinations and fluorescent expression was determined over 40 days.

In some experiments mice were immunized with different vaccines (control—non-immunized animals) and then challenged with CT26.WT cells expressing *Burkholderia pseudomallei* antigens 90 days after the second immunization. In these experiments mice were challenged only with CT26.WT cells stably transfected with the bicistronic vectors expressing mCherry as a separate gene and both studied *B. pseudomallei* antigens—Hcp1 and TssM fused together. A total of 5 × 10^4^ tumor cells were injected into each mouse to minimize the effect of tumor growth on immunological responses. For each animal, the mCherry signal at the injection site was measured over time and normalized to the signal at Day 2. Most experiments were terminated at day 64 or earlier if the tumors progressed rapidly. Mice that did not develop tumors were monitored for up to 120 days. The site of vector injection was visualized using the Xenogen IVIS® Spectrum *in vivo* imaging system (Caliper Life Sciences) set to excite mCherry and eGFP.

### Vaccine and Fluorescent Reporter Vector Composition

All DNA vaccine vectors contained a pVAX1 backbone (Thermo Fisher Scientific) especially designed for high antigen expression and low risk of chromosomal integration. The platform DNA vaccine construct encoded the N-terminal of the RSV viral protein F (RSV strain A2) which contains amino acids 1–529 of the un-cleaved F0 precursor. After furin cleavage in the Golgi two amino acid chains connected by disulfide bonds were generated—a full length F2 and a truncated F1 (1–393 amino acids). The platform DNA vaccines that target *B. pseudomallei* include sequences encoding the full-length protein Hcp1 (BPSS1498; *B. pseudomallei* K96243) fused to the region of TssM that has been proven to be immunogenic in mice (23, 26) (BPSS1512; *B. pseudomallei* K96243; amino acids 191–474); these antigens were also fused with RSV-F resulting in a triple fusion vector: RSV-F/Hcp1/TssM. In all vaccine and fluorescent reporter vectors expressing TssM, Cys^102^ in TssM was replaced by Gly^102^ (26).

Two control vaccines contained the same Hcp1 and TssM sequences, but lacked the functional RSV-F domain. One control vaccine encoded only Hcp1/TssM genes and served as an intracellular expression control. The second control vaccine encoded Hcp1/TssM followed by the RSV-F signaling sequence (1–25 amino acids of RSV strain A2). To study the effect of RSV-F on antigen expression, secretion, and dispersion the mCherry sequence was used instead of the Hcp1/TssM sequence. This resulted in three additional fluorescent plasmid vectors: mCherry alone (pls-A), mCherry fused to the N-terminal ectodomain of RSV-F separated by a flexible linker (pls-C), and mCherry fused to the RSV-F signaling sequence also separated by a flexible linker (pls-B). All vectors (vaccines and fluorescent reporters) were delivered as transgenes under control of the CMV promoter and their expression was terminated by the bovine growth hormone polyadenylation [bGH-Poly(A)] signal.

### Mouse Immunizations

The DNA vaccines were injected subcutaneously into the tails of BALB/c mice. Equimolar amounts of the DNA vaccines (5 micrograms for the smallest vaccine vac-A) were injected subcutaneously in mice followed by local electroporation. A single 500 V/cm electrical impulse was applied across two electrodes over 5 μs by using the BTX ECM 830 square wave electroporator. We performed two identical immunizations per animal 30 days apart. Mice vaccinated with PBS were studied as a separate negative control group.

### Antigen Specific Antibody Assays

*B. pseudomallei* specific anti-Hcp1 and anti-TssM IgG_1_ and IgG_2a_ titers were measured in serum. U-bottom 96-well ELISA plates (Nunc Maxisorp, Denmark) were coated with 5 μg/well of recombinant proteins (rHcp1 or rTssM) in PBS overnight at 4°C. Plates were washed with PBS/0.1% Tween-20 and then blocked with PBS/5% goat serum (Gibco, Carlsbad, CA) for 4 h at room temperature. Serum samples were added at an initial dilution of 1:100 in triplicate, with 1:2 serial dilutions performed in PBS/1% goat serum. Plates were incubated for 1 h at room temperature, and then washed in PBS/0.1% Tween-20. A 1:10,000 dilution of HRP-conjugated anti-mouse IgG (Cat. No: NA931-1ML), IgG_1_ (Cat. No: 1070-05), or IgG_2a_ (Cat. No: 1080-05) was added to the plates for 1 h at room temperature. Plates were washed and developed with 3,3′,5,5′-tetramethylbenzidine (Sigma-Aldrich, St. Louis, MO) for 10 min, and stopped with 2 M HCl. The optical density of each well was measured at 450 nm on a Synergy™ 2 Multi-Mode Microplate Reader (BioTek, Winooski, VT) and processed by Gen5 software.

### Immune-Detection of mCherry in Cellular Extracts

CT26.WT cells were transfected with equimolar amounts of vectors pls-A, pls-B, or pls-C. Twelve hours after transfection the total cell proteins were extracted with RIPA buffer and analyzed by ELISA with anti-mCherry antibody (ThermoFisher, PA5-34974). The plates were washed, probed with HRP conjugated mouse anti-rabbit IgG-HRP antibodies (Santa Cruz, sc-2357), washed again and developed with 3,3′,5,5′-tetramethylbenzidine (Sigma-Aldrich, St. Louis, MO) for 10 min, and stopped with 2 M HCl. The optical density of each well was measured at 450 nm on a Synergy™ 2 Multi-Mode Microplate Reader (BioTek, Winooski, VT) and processed by Gen5 software. The obtained results were confirmed with several loading sample dilutions.

### Antigen Secretion and Binding Assay

Conditioned phenol red free medium from CT26.WT cells transfected with equimolar amounts of vectors pls-A, pls-B, or pls-C were subjected to ELISA with a rabbit anti-mCherry polyclonal antibody (ThermoFisher Scientific, Cat. No: PA5-34974) in a Synergy™ 2 Multi-Mode Microplate Reader (BioTek, Winooski, VT) and processed by Gen5 software. Medium collected from non-transfected cells was used as a background control. Some collected medium aliquots contained the released mCherry were applied to other CT26.WT cells, which were not transfected with any plasmid. After 6 h of culturing, these target cells were lifted with Enzyme-free Cell Dissociation Buffer (Thermo Fisher) and stained with an anti-mCherry monoclonal antibody conjugated to Alexa Fluor 488 (16D7; Invitrogen, Cat. No: M11239) to detect mCherry's presence on the surface of the target cells by flow cytometry. For the membrane binding assay, the crude membrane extract was isolated from CT26.WT cells. Cells from a 10 cm plate were scraped into 500 μl of PBS and transferred into a micro centrifuge tube. The lifted cells were incubated 15 min on ice and passed through a 27-gauge needle 10 times (or until all cells were lysed) using a 1 mL syringe. The extract was left on ice for 20 min and then centrifuged at 720 g for 5 min. The pellet was discarded and the supernatant was transferred into a fresh tube and again passed through a 27-gauge needle 10 times, then centrifuged at 10,000 g for 5 min (PrizmR centrifuge, Labnet International, Inc.). The pellet was discarded and the supernatant was subjected for ultracentrifugation at 100,000 g for 1 h at 4°C (Optima XPN-100 ultracentrifuge (90Ti rotor), Beckman Coulter). The pellet that contained crude plasma membranes was re-suspended in different antigen containing phenol red free mediums collected from the transfected with different plasmid cells. Each sample aliquot was divided and incubated separately at 4 or 50°C for 45 min. Finally, the samples were stained with an anti-mCherry monoclonal antibody conjugated to Alexa Fluor 488 (16D7; Invitrogen, Cat. No: M11239) and centrifuged at 100,000 g for 45 min at 4°C to precipitate the membrane fraction. The membranes were then twice washed in PBS and analyzed for mCherry presence using Synergy™ 2 Multi-Mode Microplate Reader set to read Alexa Fluor 488 (BioTek, Winooski, VT) and processed by Gen5 software. Medium collected from cells expressing intracellular mCherry was used as a control.

### Analysis of CT26.WT Cells Recovered From Tumors

Tumor Samples were obtained from surgical procedures 64 days after initial injection of CT26.WT cells expressing mCherry and both bacterial antigens from the sites of injection on the base of the tail. Surgical materials were placed in sterile tubes containing DMEM supplemented with 10% (FBS), Gentamicin Sulfate and L-glutamine. Surgical Samples were cut into 2–4 mm square sections with a razor blades and washed with the cultured medium at 37°C for at least 2 h, agitating every 15 min. At the end of incubation, the cultured medium was refreshed and left for 5 days in cell culture incubator (5% CO_2_). Cells growing from tissue sections were harvested by 0.05% trypsin/0.53 mM EDTA digestion, passed through a 70-μm nylon mesh cell strainer (Fisher Scientific) to remove clusters, counted, reseeded and cultured for additional 9 days (total 14 days after isolation). An aliquot of the originally injected CT26.WT cells and non-transfected CT26.WT cells were also cultured *in vitro* for 14 days. These cells were used as a comparison (positive) and negative controls for the cells recovered from the tumors. Cells were then analyzed for mCherry expression and their purified RNA and DNA was analysis for the presence of the Hcp1/TssM sequence by qPCR.

### Statistical Analysis

Data are expressed as mean ± SD. Changes in fluorescent marker intensity, antibody titers, antigen expression and secretion, ELISA, cytokines levels, cytotoxicity, and quantitative real-time PCR results were compared using ANOVA combined with Fisher *post-hoc* analysis, with a *P*-value <0.05 considered significant.

## Results

### Expression of *B. pseudomallei* Antigens, Hcp1, and TssM, in Engrafted Tumor Cells Leads to Activation of *in vivo* Cytotoxic Immune Response in BALB/c Mice

**Figure 1:**
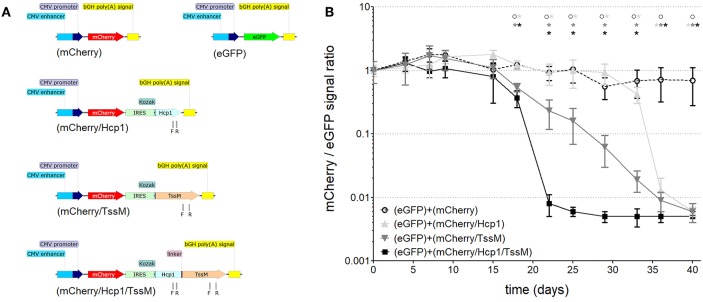
**(A)** Schematic of the DNA constructs that were stably integrated into the genome of CT26.WT cells used for studying cell-mediated cytotoxicity. CMV, cytomegalovirus; bGH poly(A), bovine Growth Hormone polyadenylation signal; IRES, Internal Ribosome Entry Site; Kozak, Kozak sequence; Hcp1, Hemolysin-coregulated protein; TssM, an immunogenic region of a secreted product of the T6SS cluster 5 encoded by the bpss1512 gene; linker, Linker sequence; F and R, positions of forward and reverse primers used for qPCR analysis. **(B)** The mCherry/eGFP fluorescent signal ratio at the site of subcutaneous injection of a mixed population of CT26.WT cells expressing eGFP and one of the four mCherry expressing vectors: mCherry, mCherry/Hcp1, mCherry/TssM, or mCherry/Hcp1/TssM. A decrease in the ratio indicates the elimination of cells expressing mCherry (*n* = 6 per group) [Asterisks (*) are color coded to match each individual animal group; *horizontal* asterisks indicate no significant difference between groups at that particular time point; *vertical* asterisks indicate a significant difference between indicated groups at a *p*-value < 0.05; open circles indicate control animals].

We first needed to determine which *B. pseudomallei* antigen(s) induced the strongest cytotoxic immune response when expressed *in vivo* in BALB/c mice. This would allow us to design the most effective DNA vector for our later immunization studies. TssM and Hcp1 have been shown to induce a cytotoxic response in previous subunit vaccine studies (21–26) so we studied them both individually and together in a BALB/c tumor model. We stably integrated one of three bicistronic vectors expressing mCherry with one or both *B. pseudomallei* antigens (Figure 1A) into CT26.WT cells (a colon carcinoma cell line that causes tumors when injected into immunocompetent BALB/c mice) using the minimal piggyBac integrative system (27). In one bicistronic vector (mCherry/Hcp1) we delivered the entire sequence for the full-length protein Hcp1 (BPSS1498; *B. pseudomallei* K96243); the second bicistronic vector (mCherry/TssM) had an immunogenic region of TssM (BPSS1512; *B. pseudomallei* K96243; amino acids 191–474); and the third bicistronic vector (mCherry/Hcp1/TssM) harbored both sequences separated by a flexible linker (Hcp1 and TssM fused together). Two vectors that expressed mCherry or eGFP alone were stably integrated into CT26.WT cells as controls.

Mice were injected with CT26.WT cells expressing eGFP plus an equal number of CT26.WT cells expressing mCherry alone, mCherry plus Hcp1, mCherry plus TssM, or mCherry plus both Hcp1 and TssM. This strategy allowed both fluorescent proteins to be expressed and visualized within the same tumor even though they were expressed in different cells. mCherry and eGFP fluorescence was determined over 40 days using a live animal imager. A total of 5 × 10^5^ tumor (CT26.WT) cells were injected subcutaneously into the tail of each mouse. Before administration, all four groups of mCherry positive CT26.WT cells were sorted several times to select cells with the same fluorescent signal. We confirmed mRNA expression of the bicistronic integrated constructs by qPCR using primers to Hcp1 or TssM. Control mice were injected with an equal number of eGFP-expressing and mCherry-expressing cells (i.e., mCherry with no antigen attached).

**Figure 2:**
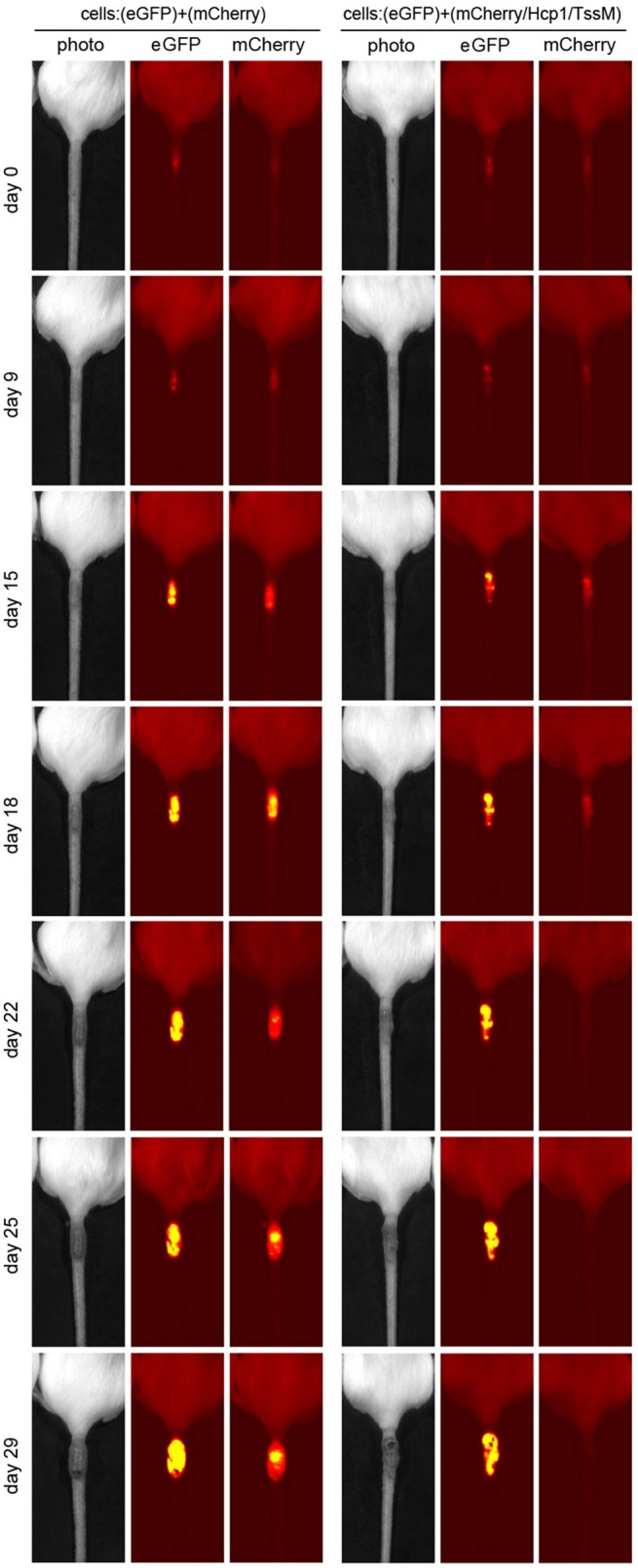
Representative tumors that developed in mice injected with a mixed population of eGFP- and mCherry- expressing CT26.WT cells **(Left)** or eGFP- and mCherry/Hcp1/TssM- expressing CT26.WT cells **(Right)** at indicated days after injection. Second label line: Photo, photographic image; eGFP, eGFP fluorescent signal; mCherry, mCherry fluorescent signal.

CT26.WT cells that expressed only mCherry or eGFP appeared to be immunologically tolerated and generated progressively larger tumors. These tumors expressed both eGFP and mCherry in equal proportions [Figure 1B (open circle) and Figure 2 (left panel)]. Mice injected with a mix of CT26.WT cells that expressed eGFP or mCherry plus Hcp1 and/or TssM formed tumors during the first 2 weeks that expressed eGFP and mCherry equally (yielding a 1:1 mCherry-eGFP ratio (*y*-axis, Figure 1B). Between 2 and 3 weeks, however, the mCherry/Hcp1/TssM -expressing cells were rapidly eliminated, leaving only eGFP-positive cells in the tumor [Figure 1B (filled square) and representative Figure 2 (right panel)]. This suggested that a cytotoxic immune response had been activated against the mCherry/Hcp1/TssM-expressing cells due to the presence of the *B. pseudomallei* antigens; in contrast a cytotoxic immune response was not generated against eGFP-expressing cells and these cells continued to grow resulting in a dramatic decrease in the ratio of mCherry to eGFP fluorescent signal.

The elimination of CT26.WT cells that expressed only a single *B. pseudomallei* antigen was delayed compared to cells that expressed both antigens, although the elimination kinetics were different for each antigen. In CT26.WT cells expressing TssM, the mCherry fluorescence slowly declined between 20 and 40 days whereas in CT26.WT cells expressing Hcp1, there was no decline in mCherry fluorescence until 35 days when fluorescence decreased abruptly (Figure 1B). These results demonstrated that while neither mCherry nor eGFP can induce a cytotoxic immune response in BALB/c mice when expressed *in vivo*, both Hcp1 and TssM do; it also indicates that the kinetics of an immune response to any single antigen expressed in target cells can vary and needs to be determined experimentally. Because the presence of both antigens together resulted in faster activation of a cytotoxic response against these tumor cells, we selected this double-antigen configuration to test several composite DNA vaccines against *B. pseudomallei*.

### A Truncated Extracellular Domain of RSV-F Fused to mCherry Facilitates Its Transfer to Other Cells

**Figure 3:**
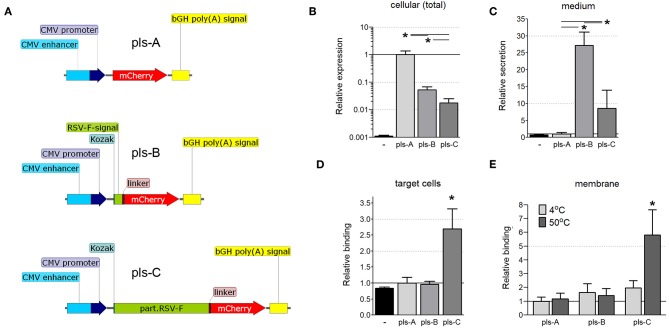
**(A)** Schematic of the mCherry-expressing DNA constructs (pls-A, pls-B, and pls-C). CMV, cytomegalovirus; bGH poly(A), bovine Growth Hormone poly polyadenylation signal; Kozak, Kozak sequence; RSV-F-signal, signaling sequence (the first 25 amino acids) of the F protein of the Respiratory Syncytial Virus; linker, Linker sequence; part.RSV-F, truncated portion of the F protein of the Respiratory Syncytial Virus (the first 529 amino acids of the F0 precursor). **(B–D)** Fusion of mCherry to the RSV-F ectodomain facilitates its transfer to neighboring cells. CT26.WT cells were transfected with plasmids pls-A (no RSV-F), pls-B (signaling peptide of RSV-F), or pls-C (entire RSV-F ectodomain). All transfected cells expressed mCherry that fluoresces and can be measured directly **(B)**; only cells transfected with a plasmid that includes the RSV-F signaling peptide (pls-B) or the entire RSV-F ectodomain sequence (pls-C) were able to secrete mCherry into the medium **(C)**; only medium from cells transfected with the entire RSV-F ectodomain encoded plasmid (pls-C) was able to transfer mCherry to non-transfected target cells **(D)**. Control (black, far left bar) represents the background signal of non-transfected cells **(B)**, the medium collected from non-transfected cells **(C)** or cells incubated in this medium **(D)** (*n* = 6, *P < 0.05). **(E)** Surrogate triggering of RSV-F fused to mCherry by mild heating increased its co-precipitation with membranes. The collected mediums obtained from cells expressing mCherry or its secretable fusion modifications after transfection with pls-A, pls-B, or pls-C were applied to the membrane fraction obtained from other (non-transfected) CT26.WT cells. A portion of this mix was kept at 4°C, while the other portion was heated to 50°C for 45 min. The membrane fractions were then centrifuge precipitated and analyzed for mCherry fluorescent using a microplate reader (*n* = 6, *P < 0.05). Results were normalized to data for pls-A **(B–D)** or pls-A at 4°C **(E)**.

We next determined whether fusing the ectodomain of RSV-F to bacterial antigens would facilitate the transfer of those antigens to neighboring cells. Three DNA vectors encoding the fluorescent protein mCherry were constructed: one in which mCherry was restricted to the cytoplasm (Figure 3A, pls-A); one in which mCherry was fused to the signaling sequence of RSV-F to allow secretion from the cell (Figure 3A, pls-B); and one in which mCherry was fused to a truncated version of RSV-F that allowed not only secretion from the cell, but also post-secretion binding to cell membranes (Figure 3A, pls-C). These vectors were tested to determine the effect of fusing RSV-F to mCherry on its expression, secretion, and post-secretion membrane binding, all considered important factors for developing a balanced immune response (28).

CT26.WT cells were transfected with equimolar amounts of one of the three DNA vectors detailed in Figure 3A. Twelve hours after transfection, the total cell protein extracts and the cultured mediums from transfected cells were tested by ELISA with a anti-mCherry polyclonal antibody. mCherry was expressed in all transfected cells (Figure 3B), but was present in the medium only when it was fused to the RSV-F signaling peptide (pls-B) or the full ectodomain of RSV-F (pls-C, Figure 3C). (The background signal of the cellular extract and media from non-transfected CT26.WT cells is shown in black). These cell culture mediums were then applied to untransfected CT26.WT cells. After 6 h of incubation, cells were harvested, stained with an anti-mCherry monoclonal antibody conjugated to Alexa Fluor 488 and analyzed using flow cytometry. We detected mCherry only in the cells that had been incubated in medium taken from cells expressing and secreting mCherry fused to the entire RSV-F ectodomain (Figure 3D, pls-C). This was true even though the amount of mCherry was significantly less in this medium than in the medium of cells that expressed mCherry fused to the RSV-F signaling sequence only (Figure 3C, pls-B). This data indicated that the ectodomain of RSV-F was able to shepherd mCherry between cells.

RSV-F anchors the virus to the target cell membrane (11), the first step in viral envelope-host cell membrane fusion. This fusion first requires that RSV-F be triggered (the translocation of the hydrophobic tail from within the hydrophilic region of RSV-F to outside the region) to facilitate insertion of the fusion peptide into the target cell membrane (13). We wanted to determine whether the ectodomain of RSV-F that we had fused to mCherry actually underwent binding to cell membranes in response to a certain stimulation or whether this interaction was due to non-specific effects. Elevated temperatures (45–55°C) can trigger six-helix bundle formation (12, 29). Therefore, we used temperature as a surrogate trigger to determine whether the truncated RSV-F ectodomain fused to another protein (mCherry in this case) would bind to cell membranes. We incubated the collected mediums obtained from cells expressing mCherry or its fusion modifications (pls-A-C) with membranes isolated from non-modified (non-transfected) CT26.WT cells at either 4 or 50°C for 45 min. The membrane fractions were then stained with an anti-mCherry monoclonal antibody conjugated to Alexa Fluor 488. As shown in Figure 3E, heating increased the fluorescence only in membranes incubated with the medium from cells originally transfected with pls-C which encoded the RSV-F ectodomain-mCherry fusion protein. These results suggested that when the ectodomain of RSV-F is fused to another protein (in this case mCherry), it retains the ability to trigger and bind to cell membranes.

**Figure 4:**
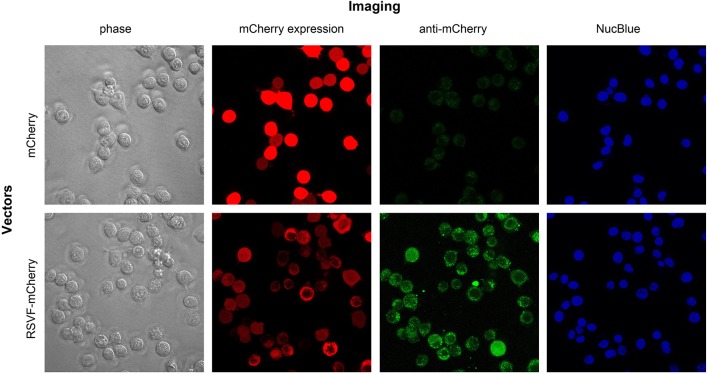
Intracellular and cell membrane-attached mCherry in CT26.WT cells transfected with pls-A (no RSV-F; top panel) or pls-C (RSV-F ectodomain: bottom panel). Imaging: phase, phase visualized cells; mCherry expression, fluorescent signal of mCherry in its native or fusion configuration; anti-mCherry, cell staining with anti-mCherry antibody conjugated to Alexa Fluor 488 (only surface protein can be visualized); NucBlue stains nuclei in viable cells.

To visualize the effect of fusing the ectodomain of RSV-F on mCherry expression and distribution, we transfected CT26.WT cells with pls-A (mCherry without RSV-F) or pls-C (mCherry with RSV-F). Forty-eight hours after transfection, live cells were directly assessed for mCherry fluorescence (“mCherry expression” panel) and also stained with an anti-mCherry monoclonal antibody conjugated to Alexa Fluor 488 (“anti-mCherry” panel) (Figure 4). Consistent with the results shown in Figure 3, mCherry was detected on the surface of cells transfected with pls-C (mCherry with RSV-F), but not on cells transfected with pls-A (mCherry only). Since the pls-C construct lacked a membrane and cytoplasmic domains on RSV-F, the presence of mCherry on the external membrane of the cells is best explained by its RSV-F-mediated ability to anchor to cell membranes post-secretion.

### Augmented Antibody Response Following Immunization With DNA Vaccine Encoding Antigens Fused to RSV-F

**Figure 5:**
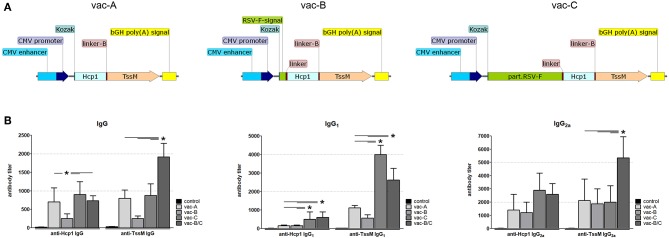
**(A)** Schematic of DNA vaccine constructs used for mouse immunization. CMV, cytomegalovirus; Kozak, Kozak sequence; Hcp1, Hemolysin-coregulated protein; TssM, an immunogenic region of a secreted product of the T6SS cluster 5 encoded by the bpss1512 gene; linker; linker-B, two diverse linker sequences; bGH poly(A), bovine Growth Hormone polyadenylation signal; RSV-F-signal, signaling sequence (the first 25 amino acids) of Respiratory Syncytial Virus protein F; part.RSV-F, truncated portion of Respiratory Syncytial Virus protein F (the first 529 amino acids of the F0 precursor). **(B)** Antibody titers against Hcp1 and TssM in the sera of immunized mice 10 days after the last immunization. Control mice were immunized with PBS. Total IgG, IgG_1_, and IgG_2a_ antibody titers against rHcp1 and rTssM are shown (*n* = 6, *P < 0.05).

We next tested if a DNA vector (vaccine) encoding RSV-F fused to bacterial antigens of interest would induce a stronger and more balanced immune response to these antigens than a vaccine encoding those same proteins without RSV-F. Using the same vector backbones depicted in Figure 3A, we replaced mCherry with the *B. pseudomallei* antigens Hcp1 and TssM (Figure 5A). BALB/c mice were vaccinated with one (or a combination) of these three DNA vectors (vac-A, vac-B, vac-C, and vac-B/C [vac-B + vac-C]). Age-matched mice vaccinated with PBS were included as negative controls. Equimolar amounts of the DNA vaccines were injected subcutaneously into the tails of BALB/c mice followed by local electroporation. We performed two identical immunizations per animal 30 days apart.

Serum from immunized and control mice was collected 10 days after the second immunization. Antibody levels (IgG) against Hcp1 or TssM were determined by ELISA using purified (recombinant) bacterial antigens (rHcp1, rTssM) to coat the ELISA plates. To determine if the tested DNA vaccines elicited a balanced Th1/Th2 immune response, we measured levels of IgG_1_ and IgG_2a_. IgG antibodies (Total, IgG_1_, and IgG_2a_) developed against rHcp1 and rTssM in all vaccinated animals, but not in control (PBS-vaccinated) animals (Figure 5B). The Th1/Th2 immune response was more balanced for TssM in all vaccinated mice compared to Hcp1 where the Th1 response was more dominant.

### Animals Immunized With RSV-F Encoding DNA Vaccines Eliminated Hcp1/TssM-Expressing Tumor Cells More Effectively Than DNA Vaccines Lacking RSV-F

After demonstrating that these DNA vaccines were able to generate a sufficient humoral response, we next determined whether they would induce a cytotoxic immune response. Mice were immunized twice (30 days apart) with the DNA vectors shown in Figure 5A or PBS (negative control). Three months after the second immunization, CT26.WT cells that stably expressed the *B. pseudomallei* antigens Hcp1 and TssM plus mCherry (the vector mCherry/Hcp1/TssM shown in Figure 1A) were injected into the tails of the vaccinated mice. As shown previously in Figure 1B, these cells initiated a strong cytotoxic immune response even in non-immunized mice. We expected that prior immunization with an Hcp1/TssM-expressing vaccine would accelerate the development of a cytotoxic immune response, leading to earlier clearance of tumor cells. To minimize the rapidly lethal effect of tumor growth, we injected 10 times fewer cells expressing the Hcp1/TssM antigen (5 × 10^4^) than we did for the experiments shown in Figure 1B. No eGFP-expressing cells were injected this time, so instead of calculating the mCherry/eGFP signal ratio we measured only the mCherry signal at the site of injection.

**Figure 6:**
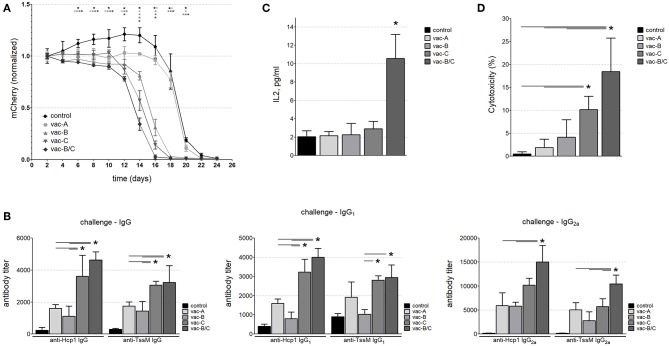
**(A)** The mCherry signal at the injection site of CT26.WT cells over time (normalized to the signal at day 2). Mice immunized with the DNA vaccine in which RSV-F is fused to the *B. pseudomallei* antigens Hcp1 and TssM (vac-C) eliminated tumor cells expressing the bacterial antigens more quickly than mice immunized with vaccines that did not include RSV-F, and non-immunized animals. Combining vac-B and vac-C [vacB/C] (Group 5, closed diamonds) resulted in the most rapid clearance (*n* = 6 animals per group) (Asterisks (*) are color coded to match each individual animal group; *horizontal* asterisks indicate no significant difference between groups at that particular time point; *vertical* asterisks indicate a significant difference between indicated groups at a *p*-value < 0.05). Antibody titers against Hcp1 and TssM **(B)** and IL-2 levels **(C)** in the sera of immunized mice 12 days after challenge with CT26.WT cells stably expressing Hcp1/TssM. “Control” represents antibody titers or IL-2 levels in non-immunized (PBS-treated) mice challenged with the same CT26.WT cells (*n* = 6, *P < 0.05). **(D)** *In vitro* cytotoxicity assay against CT26.WT cells expressing Hcp1/TssM. PBMC from the mice vaccinated with the combined vaccine vac-B/C showed the highest cytotoxic potential (~18% specific cytotoxicity). PBMC from animals immunized with DNA vaccine harboring the cytosolic antigen vac-A demonstrated the least amount of antigen specific cytotoxicity. Control, cytotoxicity of PBMC from non-immunized mice (*n* = 6, *P < 0.05).

Consistent with the results shown in Figure 1B, all mice eventually developed a cytotoxic immune response to the injected cells (Figure 6A and Supplemental Figure S1) and by Day 24, the mCherry signal was below detectable levels in all animals. However, mice immunized with DNA vaccines that contained RSV-F eliminated the tumor cells more quickly than animals that were immunized with vaccines that did not include RSV-F and animals that had been immunized with PBS (negative controls). The most rapid clearance occurred in the animals vaccinated with a combination of two DNA vectors (one that encoded the RSV-F/Hcp1/TssM fusion protein and the other that encoded a fusion protein with a signaling peptide fused to Hcp1 and TssM; vac-B/C). There was a 50% reduction in mCherry expression by around Day 13 in the mice vaccinated with vac-B/C by around Day 14 in mice vaccinated with vac-C and by around Day 15 in vac-B vaccinated animals. In contrast, a 50% reduction in mCherry expression did not occur until Day 20 in non-immunized mice or mice immunized with a vector that encoded a non-secretable version of Hcp1 and TssM (vac-A).

We assessed the antibody and cytotoxic immune response at Day 12 in a separate group of vaccinated and non-vaccinated mice. We chose Day 12 because it marked the divergence of immune responses *in vivo* and at this time point the tumor sizes were still similar in all the groups. Figure 6B demonstrates total IgG, IgG_1_, and IgG_2a_ titers 12 days after antigen-expressing CT26.WT cells were injected into immunized and control mice. All vaccinated mice generated higher antibody titers than PBS-vaccinated mice, with some domination of RSF-F formulated vaccines (vac-C, and vac-B/C). Of note, a relatively high level of IgG_1_ antibody developed against Hcp1 and TssM proteins after challenge even in the non-immunized animals; this was likely due to an antibody boosting effect of the injected cells. In contrast, there was no such increase in IgG_2a_ titers following challenge in the non-immunized mice indicating the importance of the initial immunization for the Th1 immune response to antigen.

To evaluate cytokine levels at 12 days after injection of antigen-expressing CT26.WT cells, we used a multiplex bead-based assay panel that allows for the simultaneous quantification of eight mouse cytokines secreted by Th1 and Th2 cells including IL-2, IL-4, IL-5, IL-6, IL-10, IL-13, IFN-γ, and TNF-α. There was a marked increase in IL-2 levels in mice immunized with both Vaccines B and C, but no other differences in cytokine levels were observed between vaccinated and non-vaccinated mice at 12 days (Figure 6C).

To assess antigen-specific T cell cytotoxicity against the antigens Hcp1 and TssM in vaccinated mice at Day 12, we harvested PBMCs and incubated them for 24 h with a 1:1 mix of CT26.WT cells that expressed Hcp1/TssM/mCherry (antigen-specific target cells) and CT26.WT cells that expressed only eGFP (a non-target normalization control) (mCherry/Hcp1/TssM and eGFP in Figure 1). As a control, PBMCs were incubated with equal numbers (1:1 ratio) of CT26WT cells expressing either mCherry or eGFP alone (both non-target cells) (constructs mCherry and eGFP in Figure 1A). Figure 6D shows the percentage of Hcp1/TssM/mCherry-positive CT26.WT cells that died after being co-cultured with PBMCs from each mouse group for 24 h. The results were normalized to the percentage of mCherry-positive CT26.WT cells (without Hcp1/TssM) that did not survive the 24 h co-culture. PBMC isolated from mice immunized with a vector that encoded the RSV-F ectodomain fused to Hcp1/TssM, either alone (vac-C) or, especially, in combination with the RSV-F signaling peptide (vac- B/C), eliminated CT26.WT cells expressing the bacterial antigens faster than PBMCs from other vaccinated groups and non-immunized animals.

### Tumor Recurrence in Mice

**Figure 7:**
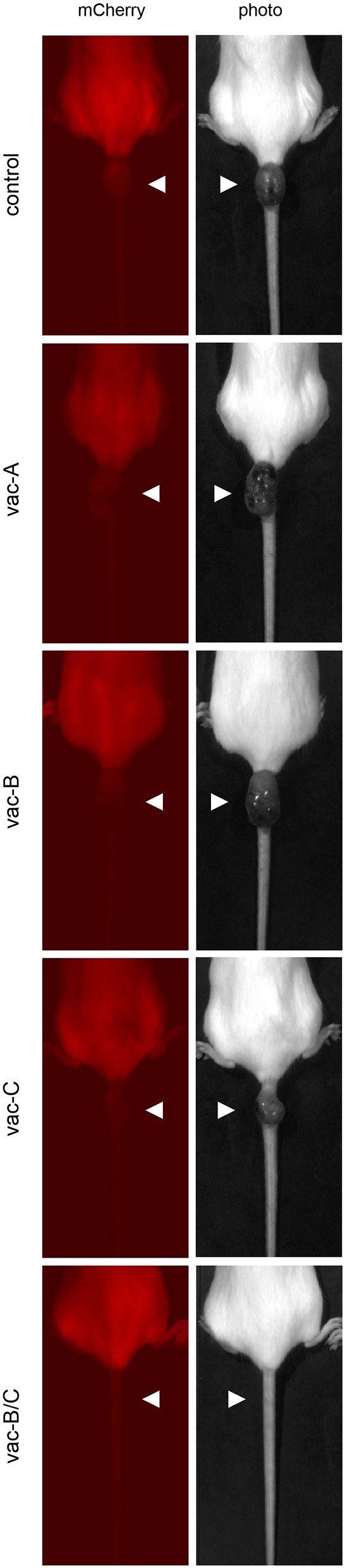
Representative images of tumors that recurred in most of the immunized animals at the site of initial injection at day 64. All mice were injected with (5 × 10^4^) CT26.WT tumor cells expressing mCherry and Hcp1/TssM. The mCherry signal at the site of injection was no longer detectable by the live animal imager in any of the mice by 24 days and there were no visible signs of tumors. Tumors recurred at the initial injection site, however, in all of the mice except in most of those initially immunized with the combined vaccine vac-B/C. The left panel demonstrates the absence of detectable mCherry fluorescence within the tumor; the right panel shows visible tumors.

As indicated in Figure 6A, mCherry expression was eliminated in all animals by Day 24. However, tumors began to recur at the same site in all animals, except those vaccinated with the combination DNA vaccine vac-B/C. Approximately 36 days after the original tumor disappeared (2 months after the initial injection), all control animals and animals vaccinated with a single DNA vector vaccine (vac-A, vac-B, or vac-C) developed large tumors at the site of injection. This was an unexpected finding. These tumors did not have any detectable fluorescent signal using the live animal imager. Figure 7 shows representative images for each group of mice 64 days after the initial injection of CT26.WT cells. Tumors recurred in all control and most vaccinated mice. In the mice vaccinated with *both* Vaccine B and C (vac-B/C), however, only 2 of 6 developed recurrent tumors whereas the other four mice remained tumor free through 120 days.

We analyzed the cells that formed tumors in these mice. Sixty-four days after the initial injection, animals were sacrificed and the tumor cells isolated and cultured in DMEM/10%FBS for 14 days (reseeded every 48 h) to ensure the removal of non-tumor cells from the cultures. An aliquot of the original CT26.WT cells expressing mCherry and both bacterial antigens that were used for the initial injection were also cultured *in vitro* for 64 + 14 days (78 days). These cells were used as a comparison (positive) control for the cells recovered from the recurrent tumors. Untransfected CT26.WT cells were used as a negative control. All cell populations were subjected to RNA and DNA qPCR analysis for the presence of the Hcp1/TssM sequence and flow cytometry to assess mCherry fluorescence.

**Figure 8:**
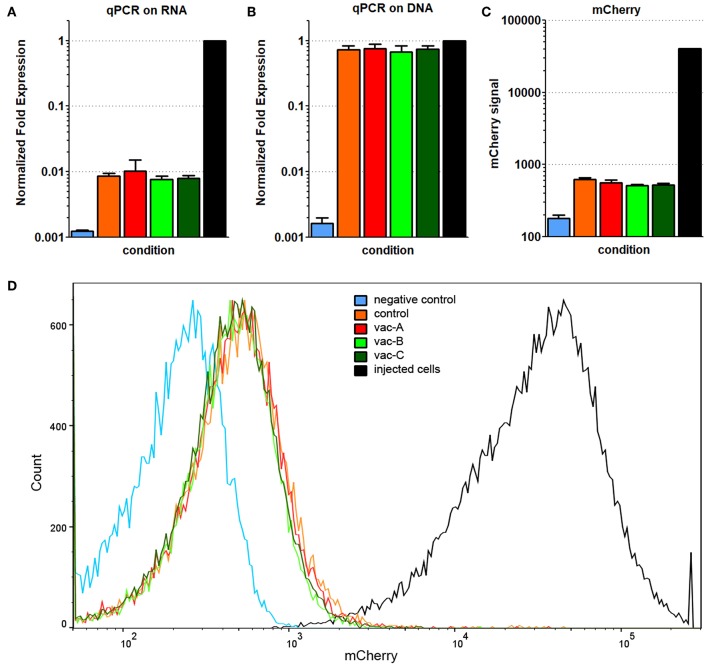
Decreased, but detectable Hcp1/TssM/mCherry levels found in cells from recurrent tumors. All recovered cell populations from the 64 day tumors were analyzed by qPCR on RNA **(A)** and qPCR on genomic DNA **(B)** for the presence of Hcp1/TssM sequences; mCherry signal was determined in the same cells by flow cytometry. Hcp1/TssM RNA sequences were markedly reduced, but not completely abolished in the recovered cells compared to the originally injected cells. In contrast, the signal for genomic DNA was very similar in the recovered and the original (positive control) cells. **(C)** mCherry expression in the recovered, originally injected, and non-transfected (negative control) cells indicate a reduction in fluorescence in recovered cells. **(D)** Representative flow cytometry data showing the reduced mCherry fluorescence, but very tight mCherry distribution profile of all recovered cells regardless of what DNA vaccine was originally use. The color code is the same in all pictures.

Expression of Hcp1/TssM RNA was still detectable in the cells harvested from the tail tumors, but it was markedly reduced compared to that in the positive control cells (Figure 8A). Consistent with that result, while mCherry fluorescence (detected by flow cytometry) was still detectable above baseline, it was reduced far below that in the positive cells (Figure 8C). The signal for genomic DNA, however, was similar between the cells recovered from the tumors and the positive control cells (Figure 8B). This suggested that a decrease in transgene expression was the cause for the decrease in Hcp1/TssM RNA expression. The low antigen expression likely allowed these cells to be tolerated immunologically giving them a growth advantage over the CT26.WT cells with higher antigen expression which were targeted and destroyed. This profile of decreased, but detectable proteins (Hcp1, TssM, mCherry) was almost identical in all recovered tumor cells regardless of the vector with which the mice was immunized (Figure 8D).

## Discussion

We describe an engrafted mouse model that uses antigen-expressing tumor cells to assess the *in vivo* cytotoxic immune response to specific antigens. We then used this model to demonstrate that a DNA vaccine in which RSV-F is fused to two antigens expressed by *B.pseudomallei*, the intracellular gram-negative organism that causes melioidosis, generates a stronger cytotoxic immune response than a DNA vaccine that lacked RSV-F.

This engrafted model capitalized on the ability of cells derived from a colon cancer cell line (CT26.WT cells) to form tumors in immunocompetent BALB/c mice. This provided the opportunity to express antigens from an intracellular organism in these “tumor” cells to determine whether we could develop an animal model of acquired cytotoxic immunity. By discriminating between weak (eGFP and mCherry) and strong (Hcp1 and TssM) immunogenic antigens, the proposed model can be used to study the cytotoxic immune response to known or unknown intracellular antigens of interest (either separately or in combination), and thus, guide the development of novel DNA vaccines against intracellular organisms. This method has the potential to be useful in situations where challenge models are not available, too expensive, or require special safety precautions, as is the case with *B. pseudomallei*.

RSV protein G (RSV-G) and RSV-F are two envelope proteins responsible for viral fusion. However, the available data suggests that RSV-G may be dispensable, and RSV-F can, by itself, bind multiple host cell receptors, such as ICAM-1, heparin, annexin II, integrins, and nucleolin and initiate membrane fusion (30, 31). Most paramyxovirus F proteins can induce cell-cell fusion when expressed on the cell surface at neutral pH, leading to the formation of giant multinucleated cells termed syncytia (32). RSV can directly infect immune cells, such as macrophages, B cells, CD4+ T cells, and CD8+ T cells (33–35). Removing the transmembrane and cytoplasmic domains of RSV-F prevents it from adhering to the cell membrane as it is secreted in pre-triggered form, making it soluble. A soluble form of RSV-F can be covalently linked with molecular tags and still retain the ability to form trimers and fuse to target membranes after triggering (12). An advantage of this technology is that delivery is not specific to certain surface molecules on professional antigen presenting cells used in alternative DNA vaccine approaches (36, 37). This might initiate a broader spectrum of cytotoxic immune responses such as antibody-dependent cellular toxicity or activation of complement (38, 39). Adding antigens of interest as the molecular tags is a simple and suitable way to deliver antigen(s) of interest to target cells. We used this modified RSV-F ectodomain to extend the immune signal of a DNA vaccine encoding the *B.pseudomallei* antigens Hcp1 and TssM.

The main potential advantage of DNA vaccines over subunit vaccines is their ability to stimulate both a humoral immune response and a cytotoxic immune response (9). This is because the antigens they encode are transcribed within the host cell and its peptides presented as a MHC I complex on the cell membrane regardless of whether antigens are secreted or not (2–6). If antigens are secreted, it can activate a broad spectrum of immune responses by direct interaction with professional immune cells or indirectly through targeting non-immune cells. The goal of linking RSV-F to the antigens was to increase the number of cells that could present them on MHC complexes thus generating a stronger cytotoxic immune response. Consistent with this hypothesis, animals vaccinated with a DNA vector encoding RSV-F fused to Hcp1 and TssM eliminated CT26.WT tumor cells expressing these antigens more quickly than mice that were vaccinated with a DNA vector encoding the same antigens, but lacking RSV-F. The presence of RSV-F in the vaccine accelerated the elimination of cells by 6 days, which decreased the time to clearance by about 25%. In addition, peripheral blood mononuclear cells harvested from mice vaccinated with the RSV-F ectodomain fused to Hcp1 and TssM had up to a 10-fold increase in cytotoxicity against antigen-expressing CT26.WT cells *in vitro* compared to PBMCs from mice vaccinated without RSV-F.

One unexpected observation was the recurrence of tumors in all the mice except those vaccinated with the combined vac-B/C DNA vector. Within 2 months after the initial injection of tumor cells (about 30 days after tumors were initially cleared based on visual observation and loss of signal from the whole animal imager), tumors recurred at the site of initial injection. These tumors were present visually, but did not express sufficient mCherry to be detected by the whole animal imager. When the cells comprising the tumors were analyzed, they expressed levels of antigen RNA that were about 100 times less than that of the initially injected CT26.WT cells expressing Hcp1 and TssM, yet had the same amount of antigen-coding DNA. This result suggests the presence of an antigen threshold in these cells below which the immune system cannot identify and target abnormal cells, thus, providing them with a survival advantage. When vaccinated with two DNA vectors, however, one encoding Hcp1/TssM with the full RSV-F ectodomain, the other encoding Hcp1/TssM with only the secretory peptide of RSV-F, these cells were targeted and destroyed. Combining the two vaccines appeared to lead to a heightened immune surveillance in these mice. This may have been due to a more thorough induction of the humoral and cytotoxic immune response, possibly through increased activation of helper T cells, but additional mechanistic studies will be needed to clarify this.

These results suggest that it is possible to engineer a DNA vaccine that generates both a strong antibody response and a strong cytotoxic response following immunization. The ability of RSV-F to facilitate secretion of the translated antigen(s) from initially transfected cells can provide enough circulating antigens to generate a humoral response. RSV-F's ability to fuse to target cell membranes, expands the number of cells that can present these antigens leading to an augmented cytotoxic immune response. Combining two different DNA vectors into a single immunization led not only to a more rapid *initial* elimination of antigen-expressing tumor cells, but also to elimination of tumor cells with low levels of antigen expression. The use of this engrafted model may be able to identify which combination(s) of DNA vectors/vaccines would be expected to be most effective at inducing a cytotoxic response that could target both high and low-antigen expressing cells, a strategy that could be useful against organisms, such as *B. pseudomallei*, that can cause both acute and latent infections. Whether the cytotoxic response against these antigen-expressing tumor cells in immunized mice translates into a similar cytotoxic (and protective) response in infected host target cells is not clear. A live challenge model will be necessary to determine the vaccine's protective effect against acute and chronic infections with *B. pseudomallei*.

## Data Availability Statement

The datasets generated for this study are available on request to the corresponding author.

## Ethics Statement

The animal study was reviewed and approved by The University of South Alabama Animal Care and Use Committee.

## Author Contributions

VS, BF, and VB conceived and designed the experiments. VS and BF performed the experiments. VS, RB, and BF analyzed the data and wrote the manuscript.

### Conflict of Interest

VB was employed by Emergent BioSolutions Inc. The remaining authors declare that the research was conducted in the absence of any commercial or financial relationships that could be construed as a potential conflict of interest.

## Supplementary Material

Figure S1 Representative images of mCherry positive tumors at sites of cell injection (white triangles) at indicated time points for experiments described in Figure 6A. All mice were injected with (5 × 10^4^) CT26.WT tumor cells expressing mCherry and Hcp1/TssM. The mCherry signal at the site of injection was no longer detectable by the live animal imager in any of the mice by 24 days. The left panel show visible tumors at days 12. Other panels show mCherry fluorescence within the tumor between days 12 and 22.
