# Supplementary figures and images for: A DNA Vaccine in Which the RSV-F Ectodomain Is Covalently Linked to the Burkholderia pseudomallei Antigens TssM and Hcp1 Augments the Humoral and Cytotoxic Response in Mice
Source: Front Immunol. 2019 Oct 11;10:2411. doi: 10.3389/fimmu.2019.02411 (PMC6797551; doi:10.3389/fimmu.2019.02411)

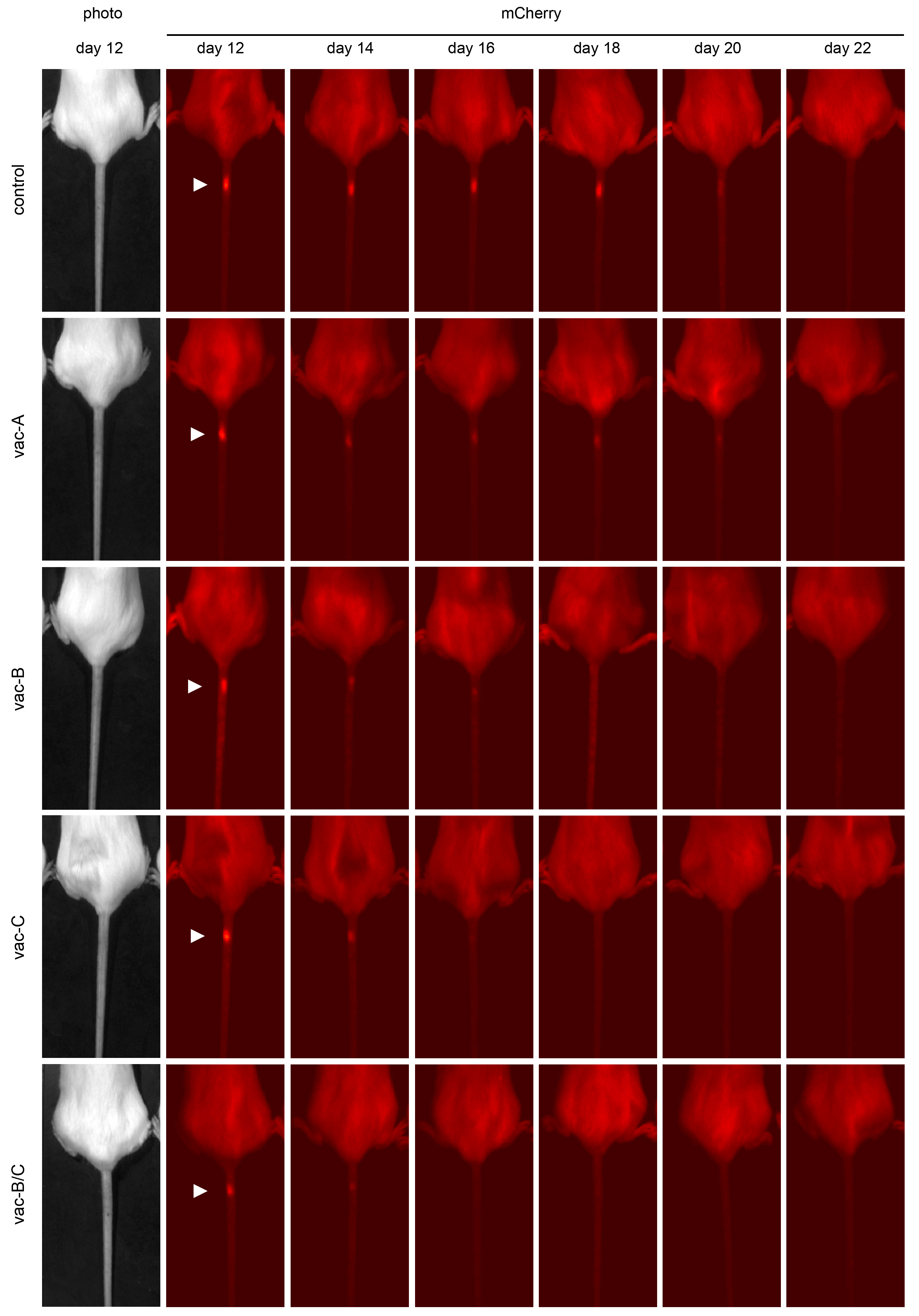

Supplement: Figure S1 — Representative images of mCherry positive tumors at sites of cell injection (white triangles) at indicated time points for experiments described in Figure 6A. All mice were injected with (5 × 10^4^) CT26.WT tumor cells expressing mCherry and Hcp1/TssM. The mCherry signal at the site of injection was no longer detectable by the live animal imager in any of the mice by 24 days. The left panel show visible tumors at days 12. Other panels show mCherry fluorescence within the tumor between days 12 and 22. [file Image_1.JPEG]
